# Intraoperative Catastrophes during Robotic Lung Resection: A Single-Center Experience and Review of the Literature

**DOI:** 10.3390/life13010215

**Published:** 2023-01-12

**Authors:** Beatrice Manfredini, Carmelina Cristina Zirafa, Gaetano Romano, Elena Bagalà, Claudia Cariello, Federico Davini, Franca Melfi

**Affiliations:** 1 Division of Thoracic Surgery, Department of Medical and Surgical Sciences, University of Modena and Reggio Emilia, 41121 Modena, Italy; 2 Minimally Invasive and Robotic Thoracic Surgery, Surgical, Medical, Molecular, and Critical Care Pathology Department, University of Pisa, 56126 Pisa, Italy; 3 Department of Anesthesia and Critical Care Medicine, Cardiothoracic Anesthesia and Intensive Care, University Hospital of Pisa, 56124 Pisa, Italy

**Keywords:** robotic surgery, intraoperative catastrophes, lung anatomic resection

## Abstract

Background: Robotic surgery is increasingly used in the treatment of lung disease. Intraoperative catastrophes, despite their low incidence, are currently a critical aspect of this approach. This study aims to identify the incidence and management of catastrophic events in patients who underwent robotic anatomical pulmonary resection; (2) Methods: Data from all patients who underwent robotic anatomical pulmonary resection from 2014 to 2021 for lung disease were collected and analyzed. Catastrophic intraoperative events are defined as events that demanded emergency management for life-threatening bleeding, with or without undocking and thoracotomy; (3) Results: Catastrophic events occurred in seven (1.4%) procedures; all of them consisted of vascular damage during lobectomy. Most of the catastrophic events occurred during left upper lobectomies (57%). Patients in this group had a higher ASA class and a higher pathological stage compared to the control group; (4) Conclusions: Intraoperative catastrophes are unpredictable events which also occur in experienced surgical teams. Given the widespread use of robotic surgery, it is essential to develop well-defined crisis management strategies to better manage catastrophic events in robotic thoracic surgery and improve clinical outcomes.

## 1. Introduction

In recent years, published trials and meta-analyses [1,2,3,4,5] have shown the safety and feasibility of the robotic approach in the treatment of lung cancer.

Robotic surgery is confirmed as a viable option for the surgical treatment of pulmonary lesions in consideration of the achievable quality of surgery and radicality of resection. The impressive technical features combined with the advantages of being a minimally invasive technique make the robotic system a valuable instrument to approach even more advanced stages of lung cancer with safety and effectiveness.

Many studies evaluated postoperative outcomes of lung cancer patients who underwent robotic surgery, comparing these results with those observed after VATS (video-assisted thoracic surgery) and open surgery. Robotic surgery seems to be associated with lower hospitalization and rate of complications, with an improved quality of life of patients [6,7,8].

Although the data regarding the positive surgical and oncological results of this approach are increasingly consistent, there is still a lack of available data on the management of intraoperative emergencies.

As we know, the robotic technique involves the surgeon operating away from the operating table, in close collaboration with the scrubbed assistants. For this reason, the coordination of the entire surgical team becomes essential to manage intraoperative catastrophes, such as major bleeding.

Many studies have been published about the management of catastrophic intraoperative complications during video-assisted thoracoscopic surgery [9,10,11,12]. Most of them conclude that it is necessary to develop specific protocols for the correct management of these situations.

There are, however, few studies in the literature about complicated intraoperative events during robotic surgery [13,14,15]. Given the technical characteristics of the robotic approach, it is necessary to increase the data concerning these scenarios, which could represent one of the critical issues of this approach.

The aim of this study is to analyze the incidence and the management of intraoperative catastrophes in patients who underwent robotic anatomic lung resection for a pulmonary tumor in a high-volume center in order to assess possible risk factors and improve the management of these events.

## 2. Materials and Methods

### 2.1. Patient Selection, Criteria, and Data Collection

The study design, the patient enrolment method, and the data collection methods were reviewed and approved by the institutional review board (ID of the Ethics Committee: 19211). Each patient was adequately informed and had to sign the informed consent.

Between January 2014 and December 2021, data from consecutive patients who underwent planned robotic anatomic lung resection for pulmonary lesions were retrospectively collected and analyzed.

Wedge resections were excluded.

All conversions to thoracotomy for reasons other than intraoperative catastrophes, such as oncological or technical reasons, were excluded.

Catastrophic intraoperative events are defined as events that demand emergency management for life-threatening bleeding, with or without undocking and thoracotomy.

Baseline patient characteristics such as gender, age, BMI (body mass index), respiratory function, and ASA (American Society of Anesthesiologists’ classification of Physical Health) class were collected and analyzed.

Characteristics of underlying lung disease such as histology, pulmonary localization, and pathological stage were also collected and analyzed.

Lastly, operative time (from the time of skin incision to the time of skin closure, including docking of the robotic system), surgeon experience, intra- and postoperative complications, morbidity, and mortality were collected and analyzed.

All features were then compared between patients with and without catastrophic events.

### 2.2. Surgical Technique

Robotic lung resections have been performed by using the da Vinci surgical system Xi^®^ (Intuitive, Sunnyvale, CA, USA) since 2015, while the da Vinci surgical system Si^®^ was used before 2015. All procedures were performed under general anesthesia, with single-lung ventilation.

The patients were positioned in the lateral decubitus, with the operating table flexed at the tip of the scapula, 5 degrees tilted posteriorly.

The robotic port mapping provided four centimetric surgical incisions for a totally endoscopic procedure using carbon dioxide insufflation (CO_2_), usually at 5 mmHg. Port standardized mapping implies the 1st surgical port (30-degree 3D 8 mm endoscope) is placed in the VII-VIII intercostal space, the 2nd and 3rd ports are posteriorly placed in the same intercostal space of the camera port at a distance of about 5 cm. The last incision is anteriorly performed just above the diaphragm, usually in the VI-VII intercostal space [4].

The hilar elements are exposed, and the broncho-vascular structures are prepared and selected. Usually, 2 graspers (fenestrated bipolar forceps, Cadiere forceps or Prograsp forceps) are employed to mobilize the lung parenchyma to better expose the hilar elements, and the dissection of the structures is also achieved by using a cautery, such as the permanent cautery hook or the Maryland bipolar forceps.

A chest drainage 28F is applied at the end of the surgical procedure.

### 2.3. Statistics

This is an observational study. Categorical data were described by absolute and relative (%) frequency and continuous data by mean and range.

## 3. Results

### 3.1. Patient Characteristics and Outcomes

Between January 2014 and December 2021, 486 patients underwent planned robotic pulmonary resections for lung tumors.

Most of the patients (460 patients, 95%) underwent surgery for malignant tumors, including primary lung tumors, metastases, and lymphomas.

Patient characteristics and surgical and clinical outcomes are listed in Table 1 and Table 2.

All included surgical procedures were anatomical pulmonary resections, in detail: 420 (86%) lobectomies, 59 (12%) segmentectomies, 4 (1%) bilobectomies, and 3 (1%) pneumonectomies (Table 2).

In seven (1.4%) cases, a catastrophic event occurred during the operation. All these events were related to vascular injury. In the analysis of the intraoperative details, all catastrophes occurred during a planned lobectomy performed by an expert thoracic surgeon.

Only one of these patients underwent preoperative neoadjuvant therapy, which was radiotherapy.

Most intraoperative catastrophes occurred during a left upper lobectomy (4, 57%); two (29%) occurred during a right lower lobectomy, and one (14%) during a right upper lobectomy.

The mean age among patients of the catastrophic group was 72 (range 65–82), slightly higher than the control group (68, range 32–86) (Table 1).

No significant differences were found between the two groups in terms of gender (male 57% in both groups), average BMI (25 in the catastrophic group and 26 in the control group), and respiratory function.

All the patients who experienced a catastrophic event had an ASA class greater than or equal to 2, and 86% of them had cardiovascular comorbidities.

The surgical time in patients who experienced a catastrophic event during the operation was on average longer than in all the other patients (322 min vs. 230 min) (Table 2).

There were no statistically significant differences in the mean hospital stay between the two groups (7 days in the catastrophe group, 6 days in the control group), while the postoperative complication rate was much higher in the catastrophe group (57% vs. 29%). The mean decrease in the hemoglobin value between pre- and immediate postoperative in the seven patients of the catastrophe group was 0.9 g/dL with a range of 0.1 to 3 g/dL. Hemoglobin levels never fell below 10 g/dL in any patient; therefore, no one required a blood transfusion. The mean intraoperative blood loss was 110 mL with a range of 50–250 mL.

No postoperative death was recorded in patients of the catastrophic group, while one patient in the control group died within 30 days after surgery.

Moreover, patients of the disaster group were characterized by a higher pathological stage (≥3) when compared to the control group (29% vs. 12.4%) (Table 1).

### 3.2. Catastrophic Events and Their Management

All the catastrophic events analyzed in this study were either intraoperative hemorrhages from the pulmonary artery or a branch of it, which occurred in six (86%) cases, or from the thoracic aorta, which occurred in one (14%) case. In five (71%) procedures, an emergency undocking and posterolateral thoracotomy was conducted for the management of the event. Compression of the site of injury was always performed immediately after the vascular disaster in order to have time to plan and manage the catastrophe. In case of emergency conversion, an initial partial undocking is suggested to perform open access, whereas a robotic instrument has to be maintained in place to compress the bleeding point until the thoracotomy is completed. Moreover, it is advisable to keep looking inside the chest with the camera until the complete undocking of the robotic system.

Below is a brief description of the details of these events and their management:

1. Planned and executed procedure: left upper lobectomy. An accidental rupture of an anterior branch of the left upper lobe artery during its dissection with fenestrated bipolar forceps was dealt with endoscopic stapler positioning;
2. Planned and executed procedure: left upper lobectomy. Principal pulmonary artery bleeding during lymph nodal dissection required undocking and posterolateral thoracotomy in attempt to control the bleeding with hemostatic, followed by tangential resection and suture of the artery;
3. Planned and executed procedure: right upper lobectomy. An accidental rupture of the small branch of the right upper lobe artery at its origin during its section with the robotic stapler took place with consequent conversion to open surgery and control of the bleeding with hemostatic;
4. Planned and executed procedure: left upper lobectomy. The tumor, in this case, was in hilar position, strongly adherent to the origin of the superior lobar bronchus. Left lower lobe artery bleeding during bronchotomy required undocking, posterolateral thoracotomy, and suture of the artery;
5. Planned and executed procedure: right lower lobectomy. Bleeding due to the accidental rupture of the right lower lobe artery during its dissection was solved by endoscopic stapler positioning;
6. Planned and executed procedure: right lower lobectomy. An accidental lesion of the thoracic aorta with fenestrated bipolar forceps during mobilization of the lung required undocking, posterolateral thoracotomy, suture, and reinforcement with a pledget;
7. Planned and executed procedure: left upper lobectomy. Accidental rupture of the dorsal branch of the left upper lobe artery during its dissection required undocking, posterolateral thoracotomy, suture, and section of the artery.

The planned surgical procedures were performed in all reported events, and no major resections were required.

**Figure 1:**
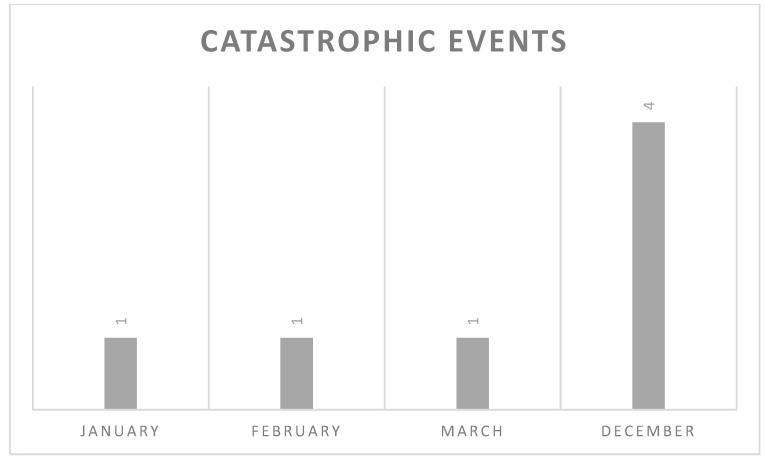
Distribution of catastrophic events through the year.

Analyzing the event distribution, most of the catastrophic events occurred in December (4, 57%) as shown in the graph below (Figure 1).

## 4. Discussion

Robotic surgery is increasingly used in the thoracic field thanks to its technical characteristics. Several studies have been published in recent years demonstrating the safety and the positive long-term results of the robotic approach in the treatment of lung cancer [1,2,3,4,5]. Moreover, it has been widely demonstrated that the robotic technique represents a valid alternative to VATS and open approaches [6,7,8] and that minimally invasive techniques lead to better perioperative outcomes when compared to open surgery [2,16].

To date, a critical aspect of minimally invasive techniques is the management of intraoperative catastrophic events, such as major bleeding.

Many studies have been published on the management of intraoperative emergencies during the VATS technique. In 2011, Flores et al. [9] evaluated their experience with thoracoscopic lung resection, reporting an intraoperative catastrophic event in 1% of cases and pointing out the necessity to implement specific management strategies to limit morbidity.

Moreover, Decaluwe et al. [10] showed an incidence of 1.5% of major intraoperative complications during video-thoracoscopic surgery in a multicenter experience.

Furthermore, Gonzales-Rivas [12] published a study about strategies for the management of different types of major bleeding during VATS surgery. The key point of this study is that any emergency must be properly assessed and that, in case of bleeding, a sponge stick must be quickly available to apply pressure to control the hemorrhage. Only once the bleeding has been temporarily controlled can it be decided whether to proceed treating the damage with or without a thoracotomy.

To date, there are fewer data in the literature on intraoperative catastrophes during robotic surgery and their management. Given the diffusion of this approach, the definition of intraoperative strategies for the management of these events is becoming increasingly urgent. A thoracotomy is usually required for the definitive management of most injuries even though there are novel techniques emerging for a minimally invasive approach.

Cerfolio, in 2016 [14], published his experience with robotic major vascular injuries, reporting a rate of 2.4%. According to this paper, the morbidity of catastrophic events can be minimized by maintaining a rolled sponge available during vessel dissection to be ready to perform compression as a fundamental first attempt to achieve hemostasis, taking time to plan the next steps, and regain calmness to act rationally and without panic.

The most common type of catastrophic event during robotic procedures appears to be pulmonary arterial injury, predominantly of the artery of the left upper lobe due to the multiple arterial branches present in this site [15]. The reported incidence of these types of injuries in robotic surgery is 0.5–2.6% [14,17,18,19,20] in series of greater than 100 robotic lobectomies.

In many series describing how to handle these alarm situations, the importance of remaining calm and in charge is emphasized. The first thing to do is to keep calm and control the bleeding with one or more pre-rolled sponges [14] or by applying pressure with a suction device [21,22].

Sakakura et al., in 2021 [23], approached the problem from another point of view: by presenting a thoroughly written emergency rollout procedure in the form of a checklist, in case of major or minor incidents. He also described how, based on their experience, conversion surgery to VATS or thoracotomy (vertical or lateral) can be performed using a new robot-assisted thoracic approach with either vertical thoracotomies or their thoracoscopic technique.

Recently, Cao et al. [13] reported the largest series of catastrophic events during robotic lung resection with a rate of 1.9%. In this study, the predictors of catastrophic events are a higher clinical stage and a lower FEV1, and the most common event was hemorrhaging from the pulmonary artery. The authors concluded that, although the incidence of intraoperative failures in robotic surgery is low, it is necessary to establish management strategies to improve clinical outcomes.

In our experience, catastrophic events occurred in 1.4% of surgical procedures, and five of seven procedures were managed with emergency undocking and thoracotomy. As already reported, all events were arterial bleedings, mainly from the pulmonary artery or one of its branches and, above all, from upper left lobectomies, in line with published data.

According to the literature, the most common factors that contributed to catastrophic events were the anatomical variants, both in terms of chest constitution and pulmonary vascular anatomy, and adherent hilar lymphadenopathies [15]. For this reason, a careful examination of the preoperative images of patients is essential to assess the anatomy and determine the eventual technical challenges in order to better plan surgery and be prepared for any eventuality.

A higher ASA class and a greater pathological stage of lung cancer characterized the patients of the catastrophic group in our study. The patient complexity could arguably represent a predisposing factor leading to the occurrence of the disaster due to the challenges that may be present during the operation. As foreseeable, the duration of the intervention was longer when the catastrophe occurred, and the patients also experienced a higher rate of postoperative complications when compared to the control group. No patient in the catastrophe group required a blood transfusion.

An interesting element that emerged from the analysis of the data is that most of the catastrophic events occurred in the month of December, probably related to the greater number of operations performed during this month and to the stress accumulated by the surgeons in the final part of the year.

Furthermore, we have not observed a reduction in the incidence of catastrophic events over the years despite the increase in surgical experience.

Seven factors played a crucial role in the management of the events reported in this study:

1. The presence of a rolled gauze nearby during the hilar dissection available to perform immediate compression;
2. The application of compression on the injury site for a few minutes, the subsequent verification that hemostasis had been obtained, and the use of the waiting time to plan the strategy to solve the disaster;
3. The leadership of the surgeon at the console and clear communication with the equipe at the table was paramount to optimize the management of critical situations;
4. The close collaboration between the surgical and the anesthesia team, which was fundamental in immediately managing the catastrophe from the hemodynamic point of view;
5. We found that in case of conversion to open surgery, it is advisable to keep a robotic grasper to compress for hemostasis control while the team at the table performs the undocking and prepares the instruments for the thoracotomy. It is also useful to practice undocking to reduce the time needed;
6. In case of conversion to thoracotomy, it is important to be able to alert an experienced surgeon, if not already present in the operating room; an expert colleague can certainly help to act rationally and without panic;
7. In conclusion, it is important to perform a debriefing with the surgical team after each procedure marked by a catastrophic event to identify critical issues and encourage ideas to improve the management of these events thus reducing their incidence.

In our experience, the predictors of catastrophic events are a higher ASA class, a higher pathological stage, and the lobectomy.

The identification of these predictor risk factors, if confirmed by a larger case study, could be useful for implementing management strategies to reduce the risk of catastrophic events.

Moreover, the development of a catastrophic case simulation program in robotic surgery could be useful in training to reduce the incidence and morbidity of these events and improve their management, putting patient safety first [24].

The limits of this study are the retrospective nature and the lack of statistically significant differences identified due to the limited number of catastrophic events observed.

## 5. Conclusions

Given the growing interest in robotic surgery, thanks to its results, it is essential to develop intraoperative strategy protocols to manage catastrophic events that may occur during operations.

The coordination of the whole surgical and anesthesia team is essential when confronting an emergency such as major bleeding.

Future studies on larger series regarding crisis management in robotic thoracic surgery are needed to better manage these events, therefore significantly reducing the need for conversion to thoracotomy and the resulting morbidity.

## Figures and Tables

**Table 1:** Characteristics of patients.

	All Patients (n = 486)	Catastrophic Event (n = 7)	No Catastrophic Event (N = 479)
Age (years)	68 (32–86)	72 (65–82)	68 (32–86)
Male	279 (57%)	4 (57%)	275 (57%)
History of smoking	393 (81%)	5 (71%)	388 (81%)
ASA class ≥ 2	462 (95%)	7 (100%)	455 (95%)
FEV1 predicted	96 (42–188)	88 (65–107)	96 (42–188)
FEV1/FVC predicted	93 (45–140)	91 (76–109)	93 (45–140)
BMI	26 (16–44)	25 (17–34)	26 (16–44)
Pathological stage			
I	278 (57%)	3 (43%)	277 (58%)
II	109 (22%)	1 (14%)	108 (23%)
III	59 (12%)	2 (29%)	57 (12%)
IV	2 (0.4%)		2 (0.4%)
Histopathology			
Adenocarcinoma	314 (65%)	3 (43%)	311 (65%)
Squamous carcinoma	77 (16%)	2 (29%)	75 (16%)
Adenosquamous	4 (1%)	1 (14%)	3 (1%)
Neuroendocrine tumor	53 (11%)		53 (11%)
Others	38 (8%)	1 (14%)	37 (7%)

**Table 2:** Intra- and postoperative outcomes.

	All Patients (n = 486)	Catastrophic Event (n = 7)	No Catastrophic Event (N = 479)
Procedure			
RUL lobectomy	154 (32%)	1 (14%)	153 (32%)
RML lobectomy	36 (7%)		36 (7%)
RLL lobectomy	81 (16%)	2 (29%)	79 (16%)
LUL lobectomy	79 (16%)	4 (57%)	75 (16%)
LLL lobectomy	70 (15%)		70 (15%)
Segmentectomy	59 (12%)		59 (12%)
Bilobectomy	4 (1%)		4 (1%)
Pneumonectomy	3 (1%)		3 (1%)
Surgical time (min)	232 (75–615)	322 (175–530)	230 (75–615)
Hospital stay mean (days)	6 (2–28)	7 (4–19)	6 (2–28)
Postoperative complications	144 (30%)	4 (57%)	140 (29%)
Prolonged air leak	84 (17%)	2 (29%)	82 (17%)
Respiratory failure	8 (2%)	1 (14%)	7 (1%)
Atrial fibrillation	19 (4%)	1 (14%)	18 (4%)
Anemization with blood transfusion	26 (5%)		26 (5%)
Pneumothorax	21 (4%)		21 (4%)
Others	8 (2%)	1 (14%)	7 (1%)

## Data Availability

The authors confirm that the data supporting the findings of this study are available within the article.
